# “Crying on the Bus”: First Time Fathers’ Experiences of Distress on Their Return to Work

**DOI:** 10.3390/healthcare11091352

**Published:** 2023-05-08

**Authors:** Suzanne Hodgson, Jon Painter, Laura Kilby, Julia Hirst

**Affiliations:** 1 Department of Nursing, Manukau Institute of Technology—Te Pūkenga, Manukau, Auckland 2104, New Zealand; 2 Department of Nursing and Midwifery, Sheffield Hallam University, Sheffield S10 2BP, UK; j.painter@shu.ac.uk; 3 Centre for Behavioural Science and Applied Psychology, Sheffield Hallam University, Sheffield S10 2BP, UK; l.kilby@shu.ac.uk; 4 Department of Psychology, Sociology and Politics, Sheffield Hallam University, Sheffield S10 2BP, UK; j.hirst@shu.ac.uk

**Keywords:** distress, return to work, fathers, fatherhood, transitions, paternal, perinatal, identity

## Abstract

There is increasing research interest in the experiences of new fathers taking paternity leave, but less insight into men’s experiences of returning to work after the birth of their first baby. For many men in the UK context, this could take place immediately after the birth or after one or two weeks of paternity leave. This paper utilizes data from a UK-based study whilst also drawing on international literature and policy contexts. A constructivist grounded theory method was adopted to generate theory from the data gathered. Twelve new fathers shared their experiences in this study by participating in audio-recorded, semi-structured interviews. This paper focuses on fathers’ experiences of negotiating the workplace as part of an overall theoretical framework related to broader transitions to fatherhood and sheds light on the distress, guilt and psychological challenges that the participants experienced when they initially returned to work. Whether fathers did or did not explicitly describe distress at this time, they all described a change in their worker identity, which for some participants led to uncertainty in the workplace. Men returning to work at this time in the postnatal period are vulnerable to experiencing distress. Flexibility and support in the workplace could be protective of their mental health. Finally, policy and practice developments are offered to support men’s transitions to fatherhood in the workplace context.

## 1. Introduction

Fathering, and men’s role in childcare, is continuously evolving [1] and there are now multiple different fathering roles and performances available to men; Cannito (p661) describes these as “several fatherhoods” [2]. The traditional role of the distant, disciplinarian provider is now less valued by men [3], with many aspiring to a more emotionally involved ideal [4], which is frequently different to the approaches of their own fathers [1]. Thus, some new fathers attempt to combine multiple parenting roles, including breadwinning, emotional support and practical care [5]. Positive paternal relationships (demonstrated by father–infant sensitivity and play) have numerous benefits including significant positive impacts on emotional and behavioural development [6,7,8,9,10,11]. Conversely, the absence of fathers can have negative consequences throughout a child’s emotional and social development [12,13].

Men who are able to take longer parental leave are found to be more involved in their children’s care, in developmental play and have closer emotional relationships than those who take less paternity leave [14,15,16,17]. Furthermore, studies have shown a positive correlation between the length of paternity leave and the extent of the positive influence on men’s lives [18]. Internationally, paternity leave provision varies considerably. For example, paternity leave policy in the USA [14] differs starkly from countries such as Sweden, Finland and Norway, whose governments offer the most generous leave to new fathers [18,19,20]. Paternity leave in these Nordic territories is considerably more than the two weeks offered in the UK and is independent of maternal leave allowances [21].

There appear to be differences in fathers’ perspectives on using and accessing leave and flexible working [14] depending on their socioeconomic or employment status. In a UK-based study, manual, semi-skilled and unskilled workers were typically found to have fewer flexible working options and access to leave than their professional counterparts [4]. Additionally, in the UK, men in higher occupational groups such as consultancy roles tend to have more access to shared parental leave, and this may be supplemented with full pay by their employers [22]. Moreover, due to the nature of their work, they also enjoy more flexible working options than fathers engaged in manual roles or the service industry, which cannot be performed from home [23]. Other researchers have found that Italian men’s decisions on whether to take paternity leave were influenced strongly by social norms, the perception that care from the mother is best for the child and, whilst the concept of involved fathering was present, it appeared to be more theoretical than something regularly enacted [24].

Returning to work can be both challenging and rewarding. Fathers wishing to remain significantly involved with their children may still face barriers such as a lack of flexibility and autonomy in their working conditions. These experiences can increase levels of stress, unhappiness and anxiety [25]. On their return to work, some fathers report a sense of dread, often wishing to have more time at home with their baby [26] and feeling sad about how much time they will spend away from their family. In the same study, however, others found a new sense of purpose in work, feeling more useful in the workplace than at home in the early days and weeks of fatherhood [26].

Research has evidenced that men can experience mental health problems related to their transitions to fatherhood [27,28,29,30]. Perinatal distress, particularly postnatal depression (PND), was, in the past, considered to be an entirely maternal problem [27]. It is however now understood that men can experience different forms of perinatal distress such as anxiety, depression and obsessive compulsive disorder (OCD). Witnessing traumatic births can also lead to anxiety, stress and relationship problems between new fathers and their partners and babies [28]. It is suggested that men manifest perinatal distress differently to women [27] and may engage in substance misuse as a form of self-medication or demonstrate anger and avoidance behaviours [31,32].

Men experience various psychological reactions during their transition to fatherhood as they try to find a role for themselves relative to their partner and as they engage in paid work, whilst navigating early parenting [33,34]. Moreover, low parenting-related self-efficacy and lower self-esteem in new and prospective fathers has been associated with paternal postnatal depression [35]. Positive intimate partner relationships have been shown to support parenting self-efficacy and parental autonomy and may be protective factors against mental health problems in both parents [36]. When mothers experience perinatal mental health problems, the risk to fathers’ mental health may increase [30,37,38]. Social support bolsters the wellbeing of new fathers, but new fatherhood has equally been shown to lead to friendship losses or changes and reductions in time for exercise, leisure and social activities [39,40]. 

Studies have shown that parental leave policy alone does not enable all fathers to co-parent their children, with deeper sociocultural and structural barriers also apparent [15,41,42]. These include traditional views on masculinity [43] and gendered norms regarding whose job it is to care for children [4,23,44]. Individually, some UK fathers are instigating changes for themselves in how they father, despite broader social and institutional norms lagging, particularly in workplace and health service policy and practice [14,45]. A recent study found that during the COVID-19 pandemic, new fathers were more able to enact involved fathering as they did not have to navigate the structural workplace barriers which consistently prevent it [46]. This same study demonstrated an association between poorer paternal mental health outcomes and what is deemed by many to be inadequate paternity leave policy in the UK [46]. 

Further research is required to better understand the factors which maximise the opportunities for new fathers to co-parent and be involved with all aspects of the care of their babies. Exploring this specifically when they return to work will help to understand the distress they experience during this specific point in their transitions to fatherhood. Furthermore, the mental health of “involved” fathers and men who take up parental leave and return to work is an under-researched topic.

This broad constructivist grounded theory (CGT) study was a doctoral research study examining all aspects of transitions to fatherhood. Different elements of the findings have applications for specific audiences (see [47] for findings relevant to healthcare practice). The current paper reports the findings with direct relevance to new fathers returning to the workplace after one to two weeks of paternity leave and is therefore likely to be of particular interest to occupational health practitioners, human resources professionals and organisational managers. The paper provides important insights into the experiences of new fathers as they navigate the UK’s current paternity leave provision and return-to-work arrangements.

## 2. Materials and Methods

Whilst there has been an increase in the study of fatherhood internationally, a study which explores the interaction between different aspects of men’s lives as they become fathers was identified as a gap in the knowledge base. To this end, a constructivist grounded theory method (CGTM) [48,49,50] underpinned by symbolic interactionism [51,52] was adopted to generate theory from the data gathered [53]. 

Approval for this study was granted by the university’s research ethics committee (HEC 2015/137 and AM/KW/D&S-311). First-time fathers with a child under one year were recruited via social media, a university website and by third sector organisations sharing adverts with their service users. Participant information sheets were emailed to the 21 respondents, with 12 men ultimately consenting to participate. Audio-recorded, face-to-face, semi-structured interviews [49] ranging from 40 min to two hours (over two sessions) were conducted between November 2016 and October 2017. Interviews generally began by asking the participants to tell their story of becoming a father for the first time. Follow-up questions sought to understand their preparations for becoming a father, experiences in the workplace, and any other topics participants highlighted as being important. 

Theoretical sampling and constant comparison processes [54] enabled the interview schedule to be adapted to explore emerging concepts and extend recruitment to include first-time fathers with a child under two years. Line-by-line coding, focused coding and theoretical coding led to the iterative formulation of the theoretical categories and final core category [55]. For further explanation of the methodological process, see [47]. CGTMs typically seek transferability rather than generalisability [48,56], hence the findings of this study may be applicable to men in different contexts or workplaces to those interviewed. 

### Participants

Twelve new fathers shared their experiences in this study, six aged 25–34 and six 35–44. Eleven identified as “White British” and one as “White Other”. All lived with their partners; six married and six co-habiting. Eleven fathers had a baby under one year and one had a child under two years. All had completed formal education, one left school at 16, another at 18, and all worked 30+ hours per week. One had a further education certificate, four were graduates and five had post-graduate qualifications. Pseudonyms have been used to protect the anonymity of participants, partners and children.

## 3. Results

These findings focus on participants’ perspectives of current paternity leave provision in the UK and, consequently, their experiences of returning to work after taking paternity leave. These experiences are often underpinned by their experiences in the healthcare setting, see [47]. Feelings such as guilt, distress and abandonment of their partner and child are shared explicitly and implicitly by the fathers, whilst worker identity is discussed in relation to feelings of uncertainty and potential insecurity. 

Participants experienced distress in the immediate postnatal hours and days and carried this with them on their return to work. These feelings relate to traumatic experiences during birth and returning home alone on the night of the birth.

“*It was scary, possibly the most scary experience that I’ve ever been through especially looking back now, it was so close to losing both of them*.”

Mark, pp. 26–27

Returning home from the hospital alone soon after the birth was particularly confronting for some participants.

“*So, I’ll go home then at four o’clock in the morning, and I think all that happened so quick and then you’re almost left on your own to deal with it really…Just sort of difficult really, feeling separated from your wife …not seeing her and then to go home but then all of that stuff to happen and then to see the sights that you’ve seen and the emotions that flood through.*”

Alex, p. 12 and p. 15

Paternity leave was discussed at length with an overwhelming sense of dissatisfaction with current policy. There was a consensus that two weeks of paternity leave was inadequate and that this paid leave should be significantly longer:

“*Two weeks of paternity leave… is ridiculously short.*”

Ethan, p. 19

Iain expands on this with his perspective on this policy:

“*I found that almost ridiculous that the man doesn’t get any time off; a week, two weeks, but it’s definitely not long enough to get used to it. Even if they did extend it to a couple of months maybe just to get used to this change in your life and then you could go back to work at least…I get two weeks and I’m thrown straight back into work and the biggest change of my life, I don’t know what I’m doing at all at that point and then I just go back to work …I’ll see you tonight for an hour.*”

Iain, pp. 29–30

On their return to work, participants expressed a feeling of abandoning their new family. There was a sense of not having had enough time to settle as a newly formed family and that inadequate paternity leave may be denying new families the opportunity to bond properly.

“*Everything got on top of me, I had to go back to work and leave the baby crying… she’s absolutely screaming…I can’t just not go to work, as much as I’d love to stay here and help you, I can’t, because I’m only allowed two weeks, that’s all you’re allowed.*”

William, pp. 24–25

Participants indicated that their role as fathers was not currently endorsed by policies related to paternity leave. They understood and articulated the consequences, including missing out on family time and on their child’s developmental milestones, and not being able to adequately support their partners. The inequality felt in relation to paternity leave was unmistakable in participants’ stories and a significant point of sadness and regret.

“*I’d not really thought about coming back to work and kind of knowing that I will miss a big chunk of his life because I am at work, I’m out of the house most of the day and thinking about, is this what it will be like for me, not forever, but for the long-term future, and I’m out of his life for more time than I’m actually in it.*”

Iain, p. 27

Participants expressed difficulty in motivating themselves when they returned to work. They questioned their impact and usefulness as paid employees when they were exhausted and distracted in the early weeks of fatherhood.

“*I remember… crying on the bus on the first day. So, a combination of just being sleep deprived and really not wanting to be there when I was at work, I felt pretty rotten.*”

Frank, p. 16

The challenges of the early weeks of new fatherhood appeared to be understood by some employers where expectations on participants’ workplace performances were lowered, at least in the initial weeks.

“*I think I’ve been lucky in that my boss, my manager at work is very understanding, kind of put me in a quiet corner for those first few weeks back.*”

Frank p. 2

In some cases, participants had very positive experiences with employers extending leave or providing compassionate leave if their babies had been unwell post birth.

“*Certainly, in the last year my work has been pretty flexible, I think, in terms of allowing me a little bit more leeway of coming maybe slightly later and maybe not working, not exactly working all my hours in a week, things like that they’ve been quite good with.*”

Alex, p. 14

Participants aspired to fulfil their roles as co-parents. A significant aspect of this was a desire to attend to their babies overnight and share such responsibilities with their partners. However, tensions arose between workplace pressures and expectations at home during the early weeks of fatherhood which compromised their desired performances as worker, father and partner.

“*I felt like I was abandoning my duties. If the baby’s crying her eyes out at ten o’clock at night and I’m up at four I’m like ‘will you see to the baby’ and you can see in her eyes she’s like kind of ‘what’? And you feel so guilty but what can you do, you know what I mean, you’re not given much choice but to live that kind of life…That feeling you can’t help somebody even though it’s part of your job as well, but you just can’t help*.”

William, pp. 25–26

These tensions were more overtly illustrated in some workplaces by explicit demands to be job focused and not necessarily care-giving focused.

“*No, you can’t be a dad because we need you to be working here and obviously you shouldn’t be trying to do this anyway, why bother with that kind of thing … yeah it’s really frustrating when you want to do things to be a better dad but you’re kind of impeded from doing that.*”

Ethan, p. 23

Tensions were present in the context of the competing responsibilities to their employer and partner. Moreover, participants described having little choice than to satisfy these demands because of their employers’ expectations. As can be seen from the quotes above, attempting to enact a fathering identity within the workplace, for some participants, was challenging.

There was an appreciation of the demands of early parenting and the work that mothers were performing. New fathers were attempting to balance their fathering role alongside workplace demands whilst also trying to support their partners by sharing the parenting load.

“*It wasn’t literally like this, but I’d come home from work and she’d basically hand the baby to me and just be like can you just take her and fine, no problem, absolutely fine, like I’d go to work, it would be intense because it was Christmas, but I’d come home and my actual job would start. That’s how I kind of see it now, it’s significantly more tiring being at home than it is being at work.*”

Fred, p. 44

Participants attempted to find other ways to balance work expectations with fatherhood responsibilities in the early weeks of their return to work.

“*It’s about managing a little bit of work to still keep a job but most of your time devoting your energy towards him for those or supporting Ann for those twelve months and that’s what my family has needed and, yeah that’s the way I’ve viewed it essentially and so my career has probably been secondary.*”

Alex, p. 18

There was also a reflection on changes in their values and attitudes towards work. Some participants changed their workplace behaviour because of becoming a father, re-evaluating their roles and responsibilities.

“*I feel like if I did act stupid and got sacked or quit then that’s having an effect on the baby because she’s not going to be able to have the things she wants and that’s not a good role model.*”

William, p. 28

For other participants, their new responsibilities at home impacted upon their professional identity which made them quickly feel out of the loop.

“*I dunno, losing a little bit of that identity about, you know, I’m kind of, kind of known at work as the film buff and someone to talk to about those things and that is kind of slowly going and people are saying, oh have you seen this film and I’m like, what’s that?*”

Albert, p. 3

In light of their experiences of their return to work, participants provided insight into the nature of support they might find or did find helpful. They spoke about this in relation to gendered stereotypes of men’s help seeking but also provided positive solutions for workplace support.

Some participants’ perspectives on men talking about their feelings were embedded within stereotypical gendered norms.

“*Don’t know if that’s just me with anxiety, or whether that’s more normal… what everyone goes through. It’s hard to tell. Cos with blokes as well, a lot of blokes keep their feelings to themselves. It’s very rare that you get the truth about what blokes are feeling, cos they like to put up a wall, as protection for themselves whereas women are a lot more emotional between each other.*”

Neville, pp. 37–38

Peer support was valued by some participants as a means of gaining support from both informal and formal sources. The importance of being able to talk to others about being a new father was shared.

“*Maybe just talking to other dads, maybe some erm, some kind of session for that, I dunno, that might be useful. Erm, yeah, new dads talking to existing dads.*”

Albert, p. 25

Opportunities to talk about feelings, on confirmation of pregnancy through to the early weeks and months of fatherhood, would be valuable.

“*I felt there were a couple of times specifically relating to some of the bad, the worries you know and the emergencies …there were a few times, I think just after that, those occasions when I would have quite liked to have been able to talk.*”

Frank, pp. 8–9

“*Well from the moment you find out right through, you know somebody you could, even just pick up the phone or go and see, right from when you find out to a few months afterwards.*”

Mark, p. 17

Overall, the participants spoke of challenges on their return to work which included tensions between home and work life. There was a strong desire to support their partners and to be involved fathers, whilst balancing their continuing role as a financial provider. Workplace behaviours changed and professional identity was challenged as a consequence of their new role as fathers being unsupported in the workplace. Participants spoke of peer support as a valuable approach to being able to talk more openly about the joys and challenges of being a new father and gaining insight and support both formally and informally.

## 4. Discussion

Negotiating the workplace during transitions to fatherhood appeared to be a source of distress for new fathers in this study. Whilst there were some examples of overt distress experienced by the participants in this study, the lack of workplace acknowledgement of men’s transitions to fatherhood may lend itself to the internalisation of distress. These findings reflect the extant literature relating to new fathers navigating workplace policies and practices including flexible working [57] and the perception of inadequate paternity leave in the UK context [58]. Fathers in other studies have identified that returning to work is both challenging and rewarding [33]. Changes in worker identity for participants were a potential source of distress, particularly for men who previously (prior to fatherhood) felt valued by others in their organisation for their knowledge, commitment and skills. In this context, the workplace could be compared to a soap opera with multiple story lines. If you are unable to watch every episode you may quickly fall behind with some of the stories, thus potentially changing your perceived status within the organisation. Recent evidence suggests that fathers requesting or taking flexible working are subjected to a “fatherhood forfeit” [59]. In this evidence, men who were balancing caregiving with paid work were viewed with scepticism and deemed to be lazy by co-workers [59]. Workplace flexibility is likely to support men to be more engaged with both the pregnancy and as involved fathers in the early weeks of parenthood. However, participants in this study and men in other studies appear to have to rely on discretionary flexibility, which is uncertain, whereas formalised flexible working should be embedded across workplace policy to promote equity [60].

As mentioned above, participants in this study described returning to work in relation to feelings of abandonment, guilt and missing out on their baby’s developmental milestones, with some indicating a lack of closeness as a consequence of this. This has been reported in other studies [25] where inflexibility in working conditions resulted in higher levels of stress, sadness and anxiety, and prohibited the enactment of caring masculinities and involved fatherhood. In the present study, when workplaces or individual managers provided a level of flexibility, new fathers were able to participate more in pregnancy-related appointments and thus felt more engaged in the process.

Traditional gendered workplaces, such as those which are male-dominated or involve manual jobs, may not afford fathers the opportunity to take time out to perform other aspects of fathering, as reflected in the current literature [15]. In these more traditional workplaces, men have been shown to be reluctant to take parental leave or to request flexible working [61,62] as they are concerned about the potential consequences on their careers and of being perceived as less masculine [57,63].

It is within the power of employers to support the development of a positive, involved father identity, by way of their respective policies and philosophies [59,60]. The pervasive presence of hegemonic masculine norms [43], a lack of flexible working opportunities and poor parental leave provision for fathers coupled with poor support for mental health creates an environment which has the potential to cause paternal distress. This is particularly concerning considering that this is a space where new fathers will spend much of their time in the early weeks of fatherhood.

As alluded to above, research suggests that gendered workplace norms heavily influence the uptake of paternity leave [15] and there is a growing understanding that parental leave policy alone is insufficient to facilitate a family’s desire to co-parent and that factors such as socioeconomics and traditional gendered norms influence these opportunities [42].

Participants in this study were open to talking to someone about how they were feeling and felt that some form of peer support would be useful. This mirrors current evidence and also grassroots practices such as Andy’s Man Club [64] and other charitable organisations such as Leeds Dads [65] and Dads Rock [66] in the UK. In previous studies, peer support has been demonstrated to be of value in supporting transitions to fatherhood [67]. Benefits described by participants in these studies include having feelings validated, reducing isolation, sharing experiences, enjoying other fathers’ company and the acknowledgement of their roles as fathers [67,68,69,70]. Validation of feelings is especially pertinent as a previous study found that new fathers felt that their feelings of distress experienced in the perinatal period were not legitimate [45].

How and whether men choose to access support is heavily influenced by traditional concepts of masculinity, and men’s help seeking takes on many forms [71,72,73]. It must not be assumed that men will access services in the same ways as women, nor that all men have the same health beliefs and behaviours [74]. Some men may prefer a male-only or male-led environment [75,76,77]. Creating spaces that are sensitive to the challenges of hegemonic masculinity and that seek to actively support new fathers in adjusting to their changing roles and identities would be a welcome start. That way, talking could be normalized, and furthermore, new fathers could be more valued and appreciated within workplaces for both their challenges and the joy and excitement of new fatherhood.

### Strengths and Limitations

The strengths of this study are reflected in its attention to the specific experiences of men navigating the workplace as they become fathers for the first time. There is growing interest in men’s experiences of paternity leave provision and flexible working, and this study offers insight into the potential distress experienced when navigating the workplace in early fatherhood. The interwoven findings of this grounded theory study have broad application to both policy and practice.

Participants’ willingness and desire to share details of their emotional wellbeing counters the common perception that men are reluctant to discuss their feelings. The helping professions may need to consider whether services are positioned to receive men’s stories and what the barriers to disclosure might be [78].

The sample of participants in this study were white, predominantly middle-class men in heterosexual relationships and over the age of 18 years. Therefore, their experiences may not be representative of fathers who identify differently from these demographic characteristics. Whilst in CGT studies, there is no requirement to be demographically representative, there is a limitation to how far these findings can be applied to other fathers, for example from non-cisgender or gay identities, different age groups, social classes, ethnicities or cultural backgrounds. Evidence does however indicate potential similarities between fathers regardless of ethnicity or cultural background [4,79,80,81] and so the findings may be somewhat applicable to other groups of fathers.

## 5. Conclusions and Implications for Workplaces and Workplace Policies

There continue to be calls for the extension and expansion of paternity leave in the UK [58] mirroring the offer in Scandinavian countries such as Sweden, Finland and Norway [21,81]. When men take up extended leave in these countries, there are benefits for the whole family [17], and fathers appear to change their perspective on work; this results in happier employees and increasing productivity and retention [81]. The benefits to infant development have been found to be significant when men are facilitated in taking parental leave or adopting shorter working hours [18]. Further research is needed to explore whether the length of paternity leave taken is associated with less distress and improved mental wellbeing in new fathers.

Whilst suggestions for policy and practice lend themselves to improved support for and acknowledgement of the needs of fathers, mothers too are likely to benefit from changes that improve workplace equity.

Since fathering is performed within the workplace, this seems an appropriate place to support the fathering roles that men want and to provide a platform for emotional support. Much more work is needed in this area of policy and development of inclusive and equal workplace philosophy; a father’s quota of dedicated and protected paternity leave such as that seen in Nordic countries may help here [81].

Recent policy changes in England have been welcomed and include paid leave for parents who have a baby in a neonatal intensive care unit [82], providing relief for fathers concerned about balancing a return to work with the distressing experience of caring for an unwell baby. However, whilst further policy change would be welcome, there are a broad range of factors which influence the uptake of paternity leave, and these must be addressed alongside any policy implementation [83]. What is well known and clearly illustrated in the present study is that men returning to work at this time are vulnerable to experiencing postnatal distress, and that flexibility in employment could be supportive of paternal mental health [25]. With this evidence in mind, we suggest the following policy initiatives.

### Policy Suggestions

- Actively promoting access to flexible working for both fathers and mothers.
- Education and awareness raising to promote parental wellbeing in the workplace and encourage an environment which is supportive and inclusive.
- Training for all levels of management and human resource departments in how to positively support new fathers in the early weeks of parenting.
- Dedicated and protected paternity leave to replace shared parental leave.
- Transparent workplace-based psychological support or mental health champions for new fathers, which aims to endorse and validate their feelings of distress and exhaustion but also their joy and pride in their new father roles.
- Workplace peer supporters for new fathers.

As part of a complete package of initiatives to support new fathers’ workplace culture, change is warranted which acknowledges the importance of involved fathers on their children’s lives, as much as mothers. Challenging workplace gendered norms may be difficult but is essential to promote equality with the potential to benefit all employees and their families.

## Data Availability

For access to data generated in this study, please contact the first author.
